# Assembling Magneto-Responsive Metal–Organic Framework Long-Range Chains

**DOI:** 10.1021/acsami.5c19158

**Published:** 2025-12-03

**Authors:** Jisoo Jeon, Valeriia Poliukhova, Hannah Y. Cook, Vladimir V. Tsukruk

**Affiliations:** School of Materials Science and Engineering, 1372 Georgia Institute of Technology, Atlanta, Georgia 30332, United States

**Keywords:** metal organic framework (MOF), magnetic zeolitic imidazolate framework, magnetic templating, field-directed self-assembly, anisotropic MOF architectures

## Abstract

Template-assisted strategies provide control over the spatial arrangement, shape, and orientation of metal–organic frameworks (MOFs). However, conventional approaches often rely on rigid templates or a soft polymer matrix to stabilize materials by adhering or wrapping MOF microparticles, which compromises the accessible porosity of MOFs and yields a random spatial organization. Here, we introduce a novel magnetic template method that produces freestanding, chain-like clusters of a 2D MOF, magnetically active zeolitic imidazolate framework (MZIF-L), on a large macroscopic scale. Preassembled magnetic nanoparticle (MNP) chains, formed under the influence of an external magnetic field, act as field-aligned nucleation centers, directing the interfacial growth of similarly oriented leaf-shaped ZIF-L platelets. Concurrent, confined growth drives interpenetration of neighboring leaves orthogonal to the chain axis while preserving consistent one-dimensional order to the macroscale. The resulting unique spiky anisotropic architectures exhibit enhanced magnetic torque and remain structurally coherent after the field removal. Embedding these chains in polyacrylamide (PAAm) hydrogels preserves their geometry in a mechanically robust matrix while maintaining long-range chain assembly and magneto-responsive actuation. Under rotating magnetic field, the MZIF-L chains exhibit synchronized rotation and helical translation, enabling rapid fluid mixing and capture of polymer microbeads through their spiky surface, which facilitates localized microplastic remediation and field-programmable materials’ micromanipulation.

## Graphical abstract

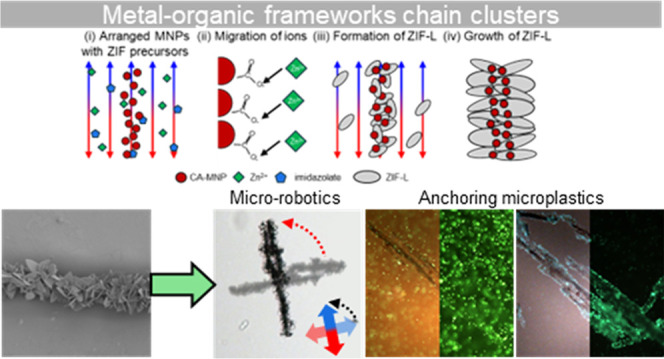

## Introduction

Formation of uniformly organized anisotropic structures at the nano- and microscale is of interest in materials research because it can lower the percolation threshold, thereby improving macroscopic mechanical properties,^1–3^ magnetic response,^4,5^ and transport behavior.^6,7^ Additionally, ordered architectures provide uniformity, directionality, and periodicity that are critical for applications in photonic crystals, metamaterials, and functional coatings.^8–10^ Such ordered architectures exhibit collective properties absent in the individual building blocks; precise control of their ordering and orientation is therefore essential for the design of advanced materials with tailored functionalities. Metal–organic frameworks (MOFs) are crystalline materials composed of metal nodes and organic linkers that assemble into periodic, open networks with a permanent porosity. Their compositional diversity and modular synthesis enable precise control over pore size, topology, and surface chemistry, making MOFs a versatile platform for constructing ordered, anisotropic superstructures.^11^ Such anisotropy in pore orientation, facet exposure, and long-range alignment is critical for sensing, catalysis, photonics, and actuation, where structural directionality and regularity strongly influence performance.^12–15^

Directed self-assembly has emerged as a powerful route to fabricate ordered architectures, leveraging entropic, interfacial, and field-induced interactions to organize crystallites with minimal external intervention.^12^ Translating the intrinsic anisotropy of MOF crystals, such as directional molecular transport and mechanical response, into the macroscopic scale requires spatial arrangement of MOF microparticles.^16^ By exploiting polyhedral crystal shapes of MOFs, tailored surface chemistries,^17,18^ and depletion-mediated interactions,^19^ researchers created diverse structures ranging from 1D chains to 2D films and 3D superlattices. These assemblies not only preserve the functionalities of individual MOF units but also exhibit emergent collective properties, including birefringence and anisotropic fluorescence.^17–19^

To support ordered MOF assemblies within soft matrices, polymer binders,^20^ such as acrylate polymers^16,18^ and polystyrene,^21^ are commonly used to bridge neighboring MOF microparticles. This approach enables the fabrication of large-area, freestanding composite films that encapsulate ordered MOF structures. However, polymeric binders can block pores and dampen the intrinsic properties of highly porous MOFs, limiting their applications. External stimuli, such as an electric field,^22,23^ can impose transient alignment of MOF particles in liquids, but the arrangement typically relaxes once the field is removed unless a binder is introduced. Consequently, binder-free directed self-assembly provides a scalable and adaptable route to preserve the MOF functionality while achieving macroscopic order.

Soft magnetic nanocomposites are widely used as stimuli-responsive media for soft actuators and robotics, owing to their rapid, programmable responses and straightforward remote control.^24,25^ Among magnetic fillers, nanoparticles, such as iron oxides, can be incorporated into polymer matrices without substantially perturbing intrinsic properties—such as mechanical or optical behavior and molecular alignment—because of their nanoscale dimensions.^26–28^ Their surfaces are easily modified, allowing for facile integration with diverse materials.^29^ Magnetic microactuators and microrobots, due to their small size and ease of control, show promise for biomedical use, including targeted drug delivery and minimally invasive surgery.^30^ However, their development often requires complex micro- and nanofabrication and frequently relies on simple polymeric hosts.^31^

Template-guided growth of MOFs offers precise control over crystal orientation and array architecture, enabling hierarchically ordered structures.^14,32,33^ Nucleation and growth on functionalized or charged templates are driven by coordination of metal ions to surface functional groups and by template-induced surface charges, which promote site-selective coordination with organic linkers and interfacial crystallization.^34^ To widen impact, the community continues to pursue more accessible fabrication routes and integration with a broader set of functional materials.^35^ Combining MOF microstructures with magnetic nanoparticles (MNPs) expands utility in areas such as biomedical delivery systems^36^ and removal of organic pollutants.^37,38^ Yet, many magnetic MOF composites are often synthesized as core–shell or particle-dispersed spherical morphologies in which superparamagnetic nanoparticles reduce cooperative magnetic alignment and responsiveness to external magnetic fields compared to larger anisotropic assemblies.

Herein, 2D MOF, specifically layered zeolitic imidazolate framework (ZIF-L), was grown in situ on prealigned MNP chain templates under an external magnetic field. This process yields directionally aligned, macroscale (several hundred micrometers long), magneto-responsive ZIF-L assemblies (named MZIF-L chain clusters, Scheme 1). In this architecture, MNP chains generate substantial magnetic torque on the ZIF-L decorated chains, enabling rapid, field-driven actuation under a dynamic external magnetic field. MZIF-L chain clusters were embedded into the polyacrylamide (PAAm) hydrogel, where the spiky surfaces of the microscopic chains mechanically interlock with the polymer network, allowing the composite to withstand deformation. In liquids, MZIF-L chains can easily rotate and translate in response to the dynamic magnetic field, enabling rapid dye mixing and the capture of polymer microbeads, which suggests applications in intermixing, spatially programmed transport, and the collection of external microplastics.

**Scheme 1:**
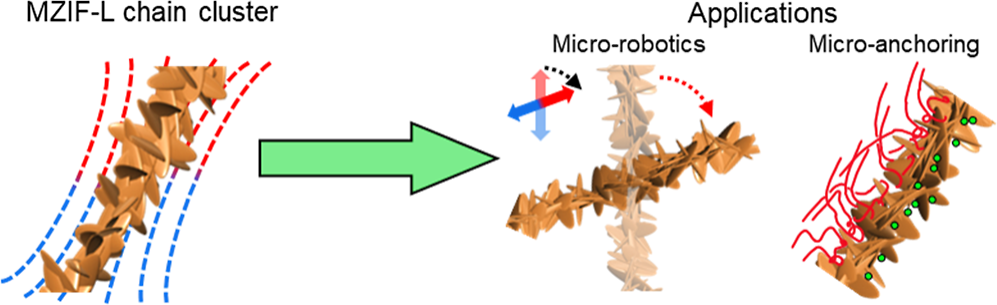
Schematic Illustration of the Formation of Large-Scale MZIF-L Chain Clusters and Their Functionalities in a Magnetic Field

## Results and Discussion

Here, we exploit the well-known formation of chain-like assemblies of MNPs, which can be consistently formed by applying a modest static magnetic field to a dilute nanoparticle suspension.^30,39^ In this approach, leaf-like ZIF-L was grown on highly ordered, preassembled long-chain carboxylic acid-functionalized MNPs (CA-MNPs) which are formed under the applied magnetic field as discussed below (Figure 1a).

**Figure 1:**
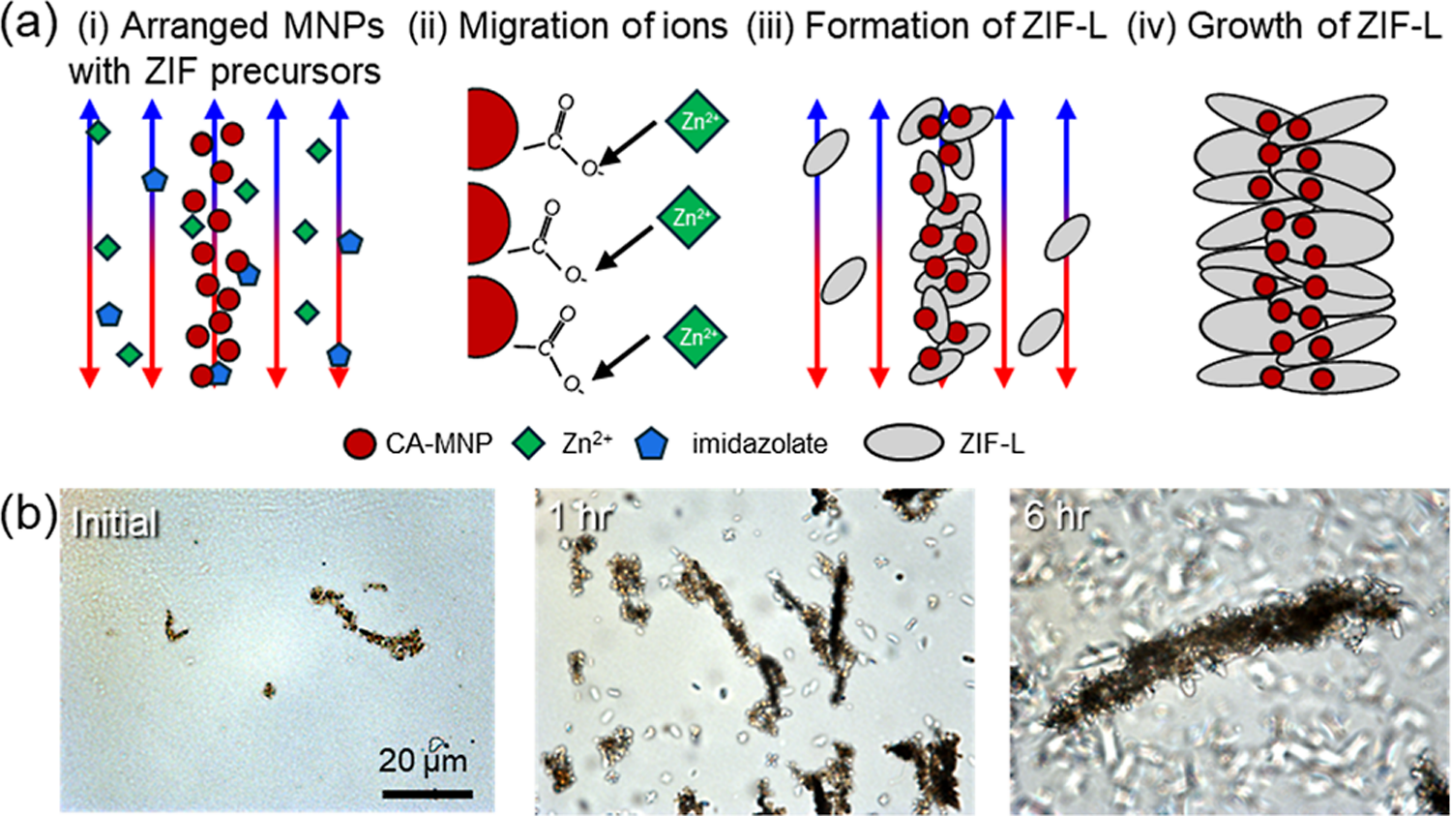
(a) Schematic illustration of the MZIF-L chain formation mechanism in aqueous solution under an applied magnetic field. (b) MZIF-L growth on MNP chains with varied time of synthesis as monitored with optical microscopy images at: (b) initial, 1 h, and 6 h. The scale bar is the same for all optical images.

To fabricate MZIF-L chain clusters, zinc ions (Zn^2+^), imidazolate linker, and 55 nm diameter MNPs dispersed in deionized (DI) water were mixed in a plastic tube (see the Methods and Figure S1). To initiate ZIF-L growth on the surface of the MNPs, the mixture was cast into a Petri dish and placed between two permanent magnets with a controllable gap allowing variation of the magnetic field strength (Figure S2). The magnet pair generated a uniform and modest magnetic field of 0.03–0.15 T depending on the distance between the magnets, promoting the formation of uniformly oriented MNP chains of various diameters in the liquid (Figure S3).

Because the MNP surfaces bear negatively charged carboxylate groups after functionalization (see Methods), positively charged Zn^2+^ ions adsorb to the MNP chain surfaces; subsequent coordination with imidazolate induces interfacial nucleation and growth of ZIF-L on the MNP chains and in the surrounding medium under the applied magnetic field; the liquid was then kept static for 24 h to allow ZIF-L to grow and fully crystallize into microscale particles on the MNP chains (Figure 1a). MZIF-L freestanding chain clusters were then isolated from ZIF-L remaining in solution via purification and separation with a magnet (Figure 1a­(iv), see Methods). The surface carboxylate groups on the MNPs provide sufficient binding sites to attract Zn^2+^ ions, as evidenced by the larger hydrodynamic radius compared to the core radius.^26^

To investigate the growth of ZIF-L platelets on MNP chains, aliquots of the reacting suspension were withdrawn at defined times during synthesis and examined via optical microscopy (Figures 1b and S4).

As we observed, within the initial 5 min of MOF formation, only original MNP chains were observed, and then, after 1 h, heterogeneous nucleation of ZIF-L is first observed along the MNP chains, rather than in the bulk suspension (Figure 1b), consistent with electrostatic preconcentration of Zn^2+^ and imidazolate near the negatively charged carboxylate surfaces and gradual linker deprotonation under the reaction conditions (Figure 1a­(i,ii)).

Subsequent growth after 1 h proceeded anisotropically, yielding the characteristic leaf-like plates that coalesced into continuous chains of magnetic nanoparticles. The chain-like magnetic template enforced ZIF-L to maintain axial registry along that direction, producing an ordered, chain-conformal shell rather than random aggregates (Figure 1a­(iii)). Finally, after 12 h of reaction time, the chain length and ZIF-L shell thickness reached a steady state, indicating saturation due to precursor depletion and self-limiting interfacial growth of MOFs and long-chain formation (Figure 1a­(iv)). These time-resolved observations support a sequence of templated heterogeneous nucleation from plate-like lateral growth to ZIF-L shell consolidation along magnetically aligned chains. The synthesized clusters are extremely stable and retain their structural integrity for more than three months after fabrication (Figure S5).

In preliminary, magnetic field-free syntheses, individual MZIF-L microparticles adopt the familiar platelet morphology characteristic of ZIF-L (Figures 2a and S6).^12,40^ Notably, when ZIF-L is grown on MNP chains, the resulting platelets exhibit slightly reduced lateral dimensions and thickness consistent with confined, template-directed growth relative to their field-free counterparts (Figures 2c and S7).

**Figure 2:**
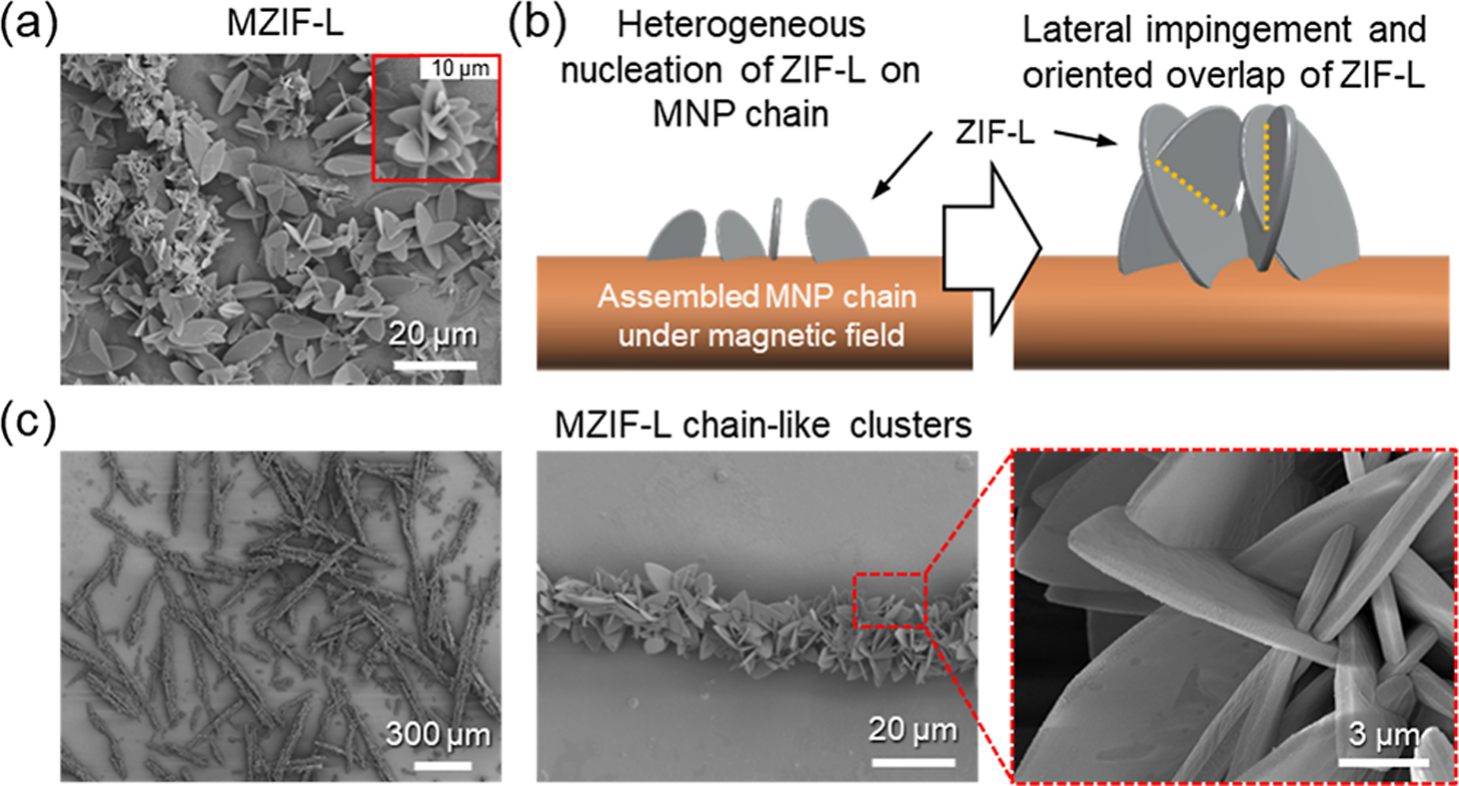
(a) SEM micrographs of MZIF-L clusters synthesized without a magnetic field, with an inset image showing small MZIF-L clusters at the beginning of chain formation. (b) Schematic illustration of heterogeneous nucleation of ZIF-L on the MNP chain surface with lateral impingement and the formation mechanism of MZIF-L chain clusters. (c) SEM micrographs of resultant MZIF-L chains after complete growth at lower magnification to show numerous long chains (left) and their interpenetrated individual morphologies at higher magnification.

SEM micrographs reveal abundant MZIF-L chain clusters extending over hundreds of micrometers, and higher-magnification images show interpenetration and overlap among neighboring ZIF-L platelets along the chains (Figure 2c). We attribute this architecture to a sequence in which (i) heterogeneous nucleation initiates at the MNP surface; (ii) anisotropic, facet-selective growth produces 2D platelets that are growing fast in-plane directions and are azimuthally biased by the 1D MNP chain template; and (iii) crowding under confined conditions drives lateral impingement and oriented overlap between adjacent leaves (Figure 2b).

This growth process results in robust, mechanically interlocked platelet chains arranged in a spiky, magnetic nanoparticle chain-guided morphology, where platelet normal vectors are orthogonal to the chain axis and the chains extend over several hundred micrometers in length, producing a large quantity, rough, high-aspect-ratio surface decorated with platelet edges and tips (Figures 2c and S8). The MZIF-L chain clusters retain a uniform, chain-conformal architecture after the magnetic field is removed primarily because the interpenetrated platelet network mechanically locks neighboring plates along the overall chain direction (Figure 2). In addition, the broad size distribution of magnetic nanoparticles does not make the rod cores porous. Rather, the heterogeneous particle sizes improve packing efficiency and reinforce the overall structural integrity of the chains.

Individual MZIF-L platelets exhibit a thickness of 200 nm and a modest in-plane aspect ratio of ∼2.6 for platelets with 4.4 μm width and 11.6 μm length on average (Figure 3a). In contrast, MZIF-L chain-like anisotropy varies systematically with the applied magnetic flux density explored in this study (Figure 3).

**Figure 3:**
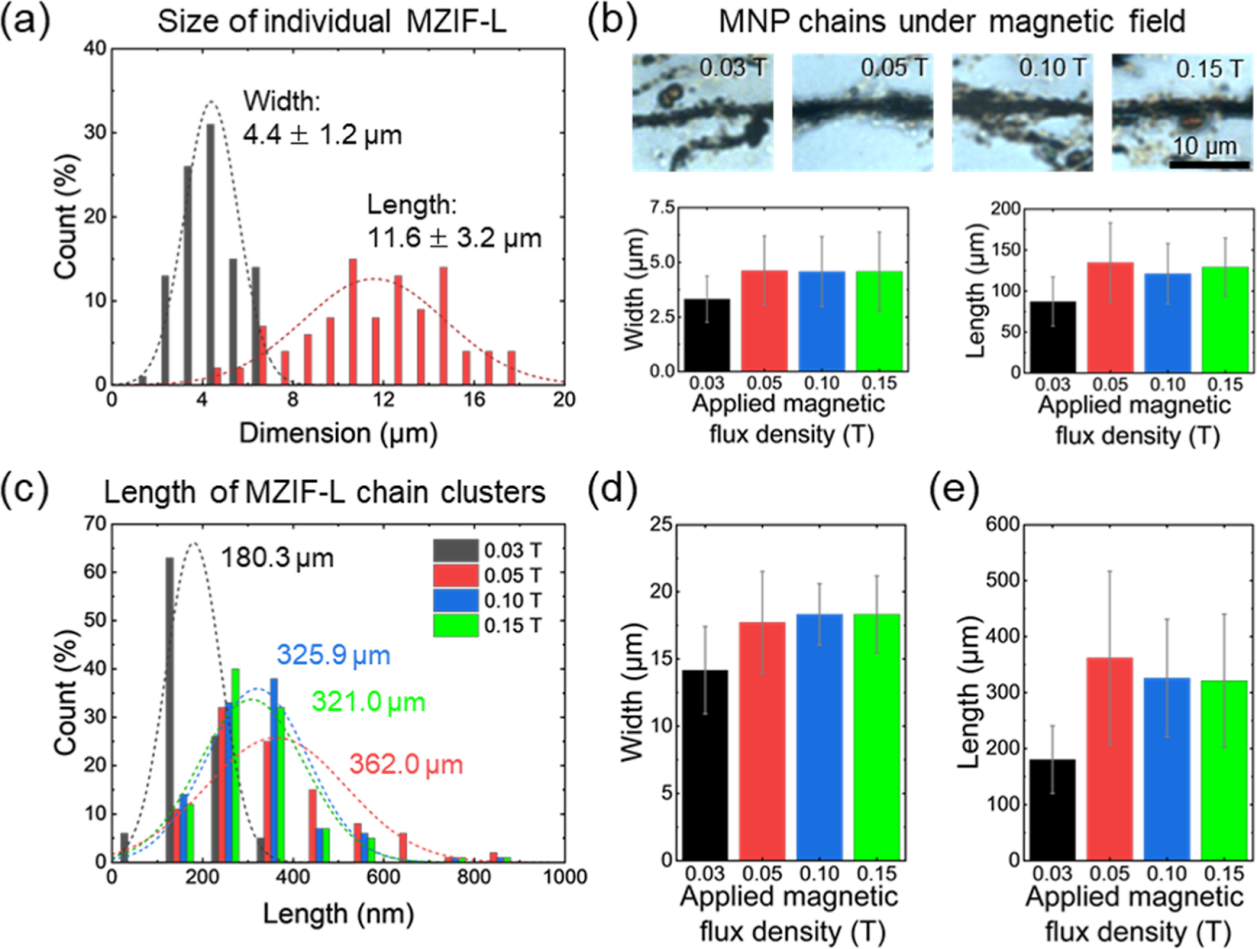
(a) Size distributions of individual MZIF-L platelets. (b) Optical micrographs of MNP chains and average sizes of MNP chains under various magnetic fields. (c) Length distribution of MZIF-L chain clusters. Average (d) width and (e) length of MZIF chain clusters. Size distributions and average sizes of MZIF-Ls and chain clusters were measured from SEM micrographs. Average sizes of MNP chains were measured from optical micrographs.

Size analysis of the MNP chains and the resulting MZIF-L chains reveals a strong correlation between their dimension changes across magnetic flux densities, consistent with ZIF-L nucleating on and ultimately encapsulating the preformed MNP chains (Figures 3c–e, and S9). Overall, increasing the magnetic flux density produces thicker and longer MNP chains reaching a saturation near the magnetic field of 0.05 T (Figure 3b). These MNP chains then act as 1D templates that set the available lateral and axial dimensions for subsequent ZIF-L growth.^41^

This templating trend is reflected by the statistics shown in Figures 3c–e and S9, where at 0.03 T, MZIF-L chains measure 180.3 ± 60.3 μm in length and 14.2 ± 3.2 μm in diameter, yielding an aspect ratio of ∼12.7. Clusters synthesized under different magnetic fields display similar dimensions (within error), with 362.0 ± 155 μm in length and 17.7 ± 3.8 μm in diameter (at 0.05 T), 325.9 ± 105 μm (length) and 18.3 ± 2.3 μm (diameter) (at 0.10 T), and 321.0 ± 118.9 μm (length) and 18.3 ± 2.9 μm (diameter) (at 0.15 T). Together, these results indicate that the morphology of the magnetic template directly governs the spatial confinement and preferential directional growth of the individual ZIF-L platelets, enabling tunable chain dimensions via the external field, which controls both the size and orientation of the MNP chains.

Furthermore, we explored whether magnetically active anisotropic fillers might offer a key advantage as sophisticated anisotropic templates. Their orientation can be directed by external magnetic fields during both assembly and chain formation. Application of a magnetic field enables the programming of filler orientation and spacing inside polymer matrices, providing a simple yet powerful approach to tune bulk mechanics via anisotropic load transfer, controlled percolation, and interfacial coupling.

To verify further this concept, MZIF-L chains were embedded in polyacrylamide (PAAm) hydrogel precursors (Figure 4a, the composite synthesis and curing conditions are provided in the Methods section).

**Figure 4:**
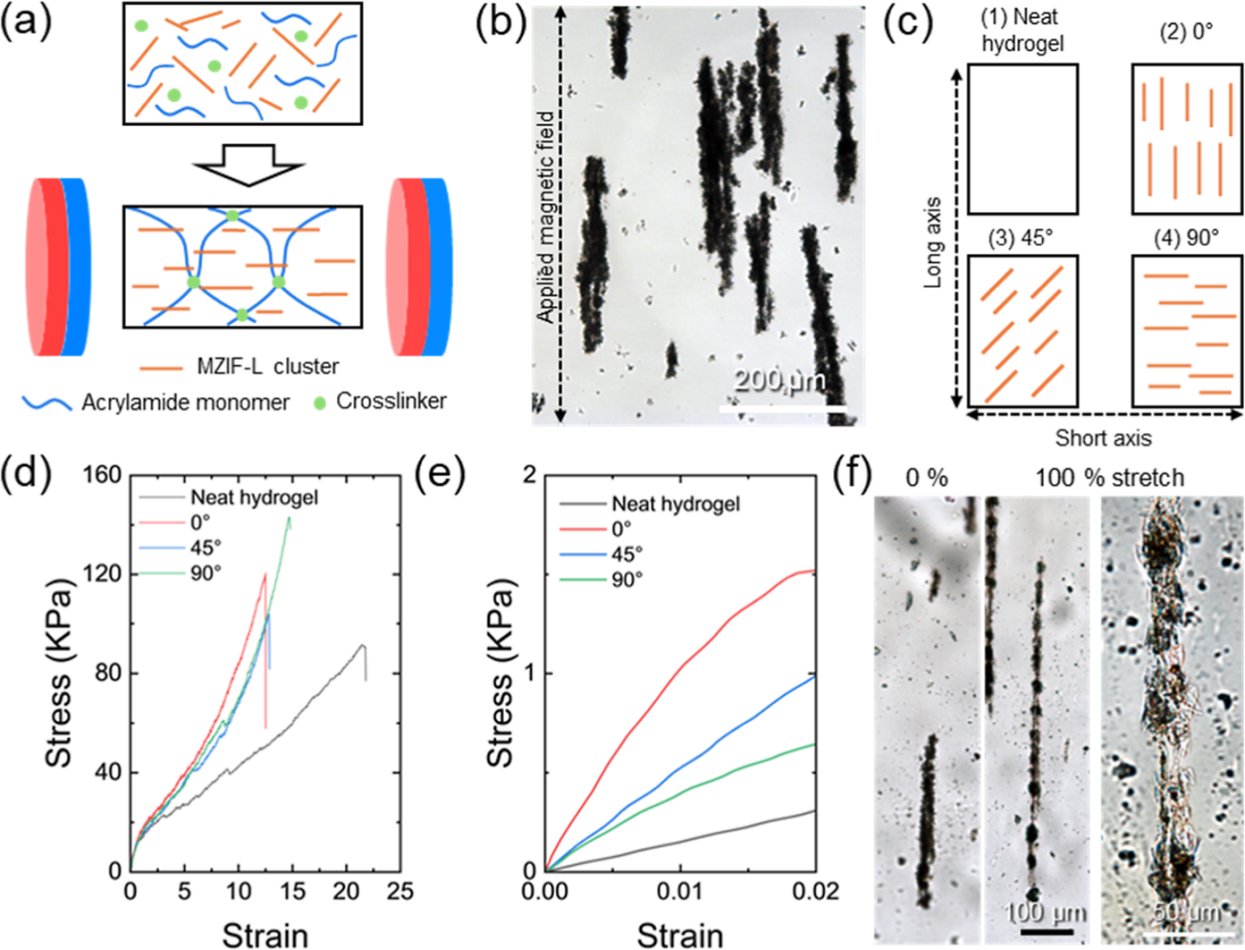
(a) Schematic of PAAm/MZIF-L chain cluster composite hydrogel synthesis under an external magnetic field. (b) Alignment of MZIF-L chain clusters under the field (magnetic flux density *B* = 0.03 T). (c) Schematic of possible arrangements of MZIF-L chains within the hydrogel. (d) Stress–strain curves of PAAm/MZIF-L composite hydrogels and (e) enlarged view of the low-strain region. (f) Optical micrographs of MZIF-L chains in the composite during uniaxial stretching, engineering strain is defined as ε = (*L* – *L*_o_)/*L*_o_ where *L* and *L*_o_ are the current and initial gauge length, respectively.

Applying a magnetic field during gelation generates a magnetic torque on the chains. Aligning their long axes before the network sets and subsequent polymerization locks in the orientation as the cross-linked network forms around the chains (Figure 4b). Four hydrogel samples were prepared to assess orientation-dependent mechanics: a neat (filler-free) hydrogel and three PAAm/MZIF-L composite hydrogels with the chain clusters aligned parallel (0°), at 45°, or perpendicular (90°) to the anticipated loading direction (Figure 4c).

During uniaxial tension, the matrix elongates while the embedded MZIF-L chains carry load; once the axial stress in a chain cluster exceeds a critical value, the brittle, intergrown platelet network fragments, which is directly observed in situ (Figure 4f). Parallel alignment (0°) maximizes the axial projection of the filler stiffness and thus improves stress transfer from the matrix to the filler, yielding a multifold increase in elastic modulus relative to the neat hydrogel (Figure 5a). Mechanistically, this improvement is aided by the bristled surfaces of filler chains, which enhance interfacial shear and mechanical interlocking.

**Figure 5:**
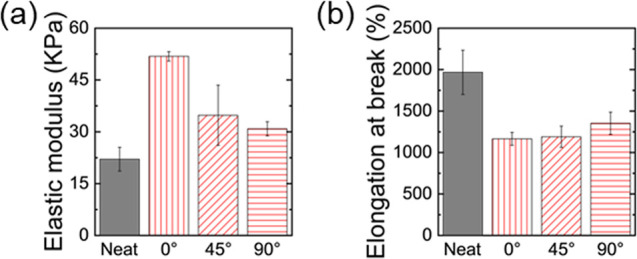
Mechanical properties of the AAm/MZIF-L composite hydrogels containing aligned chains: (a) elastic modulus and (b) elongation at break.

In contrast, 45° or 90° chain alignments resulted in a smaller modulus gain because the filler contribution is partly redirected into shear (reduced axial projection) and load transfer is less efficient. Interestingly, the highest tensile strength is observed in the 90°-aligned composite, where the MZIF-L chains are loaded largely transversely rather than axially. This reduces chain fragmentation, and the rigid platelet networks act as crack bridges, toughening the matrix (also see Figure S10). Consistent with the optical images in Figure 4f, voids nucleate at and between fractured MZIF-L chain segments; these cavities act as stress concentrators and crack initiation sites when the chains are aligned with the load, explaining the strength at 0° despite its higher stiffness.

All composites exhibit reduced elongation at break compared to the superstretchable neat PAAm hydrogel (up to 2000% strain, Figure 5b). Introducing long, highly oriented MZIF-L chains with rigid MNP cores constrains polymer chain extensibility, promoting earlier cavitation at the filler–matrix interface and localized strain around filler surfaces. As a result, the stretchability decreases to 1000% with the elastic modulus increasing by up to ∼ 3 fold, consistent with common fiber-caused reinforcement mechanisms.

Overall, these results demonstrate a structure–property relationship, where the magnetic field determines the filler orientation and orientation dictates load-transfer pathways, damage mechanisms, and crack trajectories; together, they enable tunable stiffness, strength, and ductility in magnetically programmed MZIF-L–hydrogel composites.

Because the MNPs are embedded and axially organized within each ZIF-L chain cluster, the composite chains possess a large net magnetic moment and therefore experience a high magnetic torque even under modest fields. As a result, the MZIF-L chains display rapid, coordinated rotational actuation that closely tracks the applied field direction (Figure 6a). In addition to on-axis rotation, the clusters exhibit revolving (orbital) motion, opposite to the field rotation, consistent with prior observations for rotating magnetic chains, which arises from the coupling between rotation and translation in liquid via hydrodynamic lift.^31,42^ Trajectory mapping shows spiral-like orbits of traveling clusters whose pitch depends on the field rotation rate, where at a higher rate rotationally induced lift and outward drift increase, producing broader orbits with slower inward migration; at a lower rate, inward drift dominates, and near the rotation center, the inward migration is minimal (Figure 6b and Video S1). However, MZIF-L chain clusters are easily redispersed by applying slight physical shearing to the container, which are ready to further reuse for assembly in a magnetic field (Figure S11 and Video S2).

**Figure 6:**
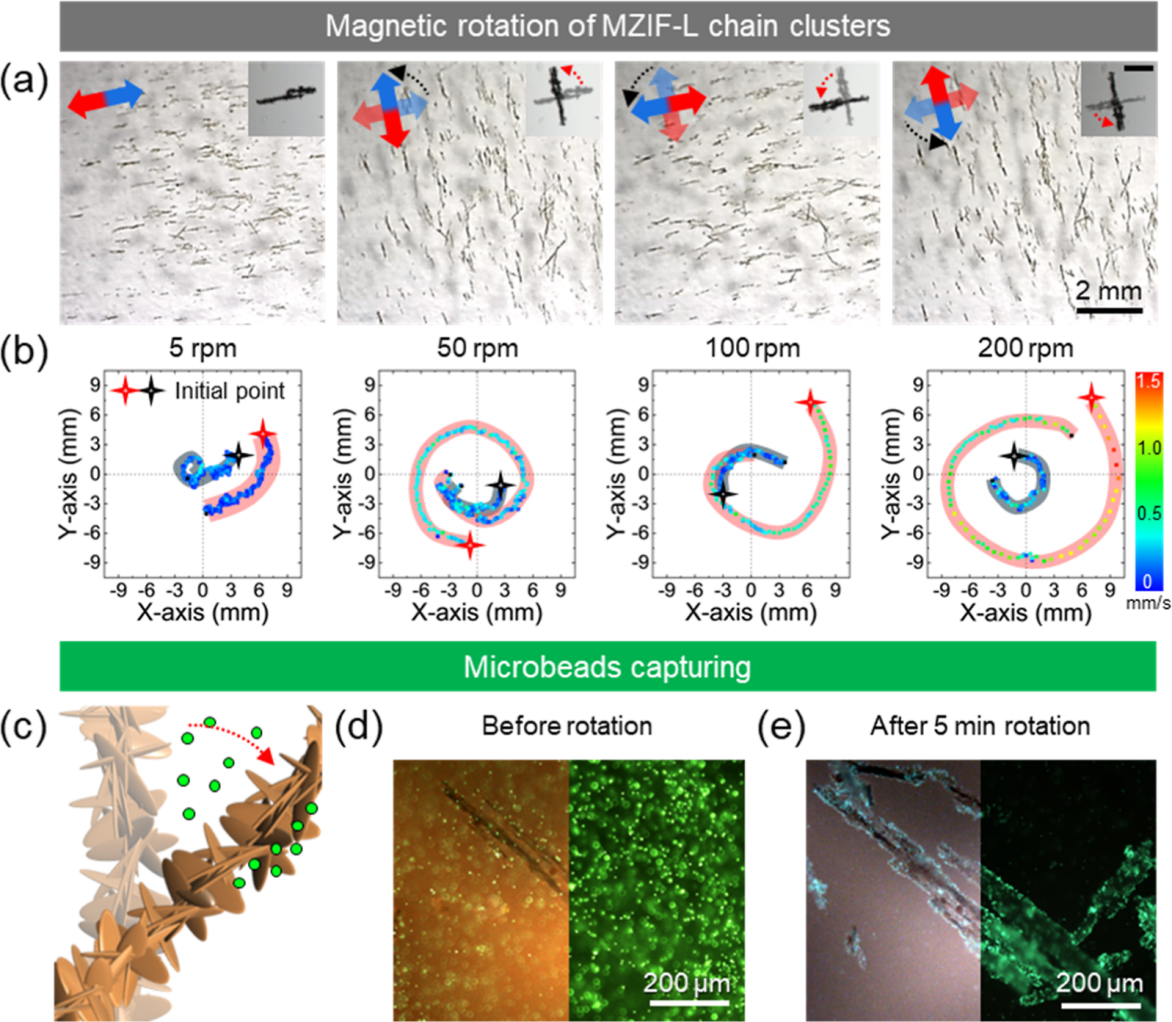
(a) Rotation of uniformly oriented MZIF-L chain clusters under a rotational magnetic field (counterclockwise); red-blue arrows denote the magnetic field direction. (b) Trajectories of the clusters’ revolving motion at different rotational rates are sampled every 0.25 s. (c) Schematic of fluorescent microbead capture by rotating MZIF-L chains. (d,e) Optical (left) and fluorescent (right) images before and after rotation showing bead capture. All MZIF-L chains in this figure were synthesized at *B* = 0.05 T. Magnetic field conditions: *B* = 30 mT and rotation rate for (d, e) is 200 rpm.

This tunable rotary*-*orbital response enables dynamic, field-controlled manipulation ability in fluids. The same magnetically driven rotation provides functional flow control over the distribution of additives, such as organic molecules and microparticles. For instance, in water, rotating MZIF-L chains generate strong local shear and chaotic advection, rapidly mixing nearby fluids: upon applying a rotating field, two initially inactive blue-dye droplets disperse and merge, mixing faster than diffusion alone, as shown in Figure S12 and Video S3. We suggest that the spiky, intergrown surface morphology of MZIF-L chains, with microscopic pockets, acts as a mechanical trap for microplastic capture (Figure 6c).

To illustrate microplastic capture, 2 μm fluorescent polystyrene microbeads were dispersed in water with MZIF-L chains (see Methods). Initially, the fluorescent beads are freely suspended and rarely come into contact with the chains (Figure 6d). After 5 min of rotation, the combination of elevated collision frequency (advection-enhanced encounters), near-surface shear, local recirculation around the bristles, and mechanical interlocking within micropockets results in robust bead attachment to the chain surfaces (Figure 6e). These field-controlled behaviors of long-range MZIF-L chain clusters point to applications in on-demand mixing, spatially programmed transport, and microplastic species, as well as diverse colloid capture in aqueous environments.

## Conclusions

In this study, we have demonstrated a template route to unique long-range chain-like assemblies of MOF microparticles by growing leaf-like ZIF-L directly on preformed, magnetically aligned MNP chains. The resulting spiky anisotropic ZIF-magnetic chains exhibit enhanced magnetoresponsivity and pronounced structural anisotropy over hundreds of micrometers. Carboxylate-functionalized MNPs (negatively charged) preconcentrate Zn^2+^ at the chain surfaces, enabling interfacial nucleation and directional growth of ZIF-L. As ZIF-L platelets reach microscopic dimensions, lateral impingement and interpenetration between neighbors produce a robust, freestanding chain-conformal intergrown platelet network with macroscopic dimensions that preserves its ordered architecture after field removal.

Beyond long-range uniformly oriented structure formation, the bristled or spiky surface with micropockets imparts dual functionality with high magnetic torque and fast field-driven actuation (rotation and controllable revolution in liquids), as well as mechanical anchoring/capture of polymers and plastic microbeads. Together, these features establish a structure–property pathway in which magnetic templating programs filler orientation and chain geometry, while the interpenetrated platelet network secures mechanical integrity and the surface topology enables on-demand mixing, transport, and microplastics capture. The demonstrated approach broadens overall MOF utility beyond conventional delivery and component separation, opening avenues in microrobotics, magnetic actuation, and dynamic microanchoring that are difficult to achieve with unstructured soft magnetic composites and MOF materials.

## Methods

### Materials

Zinc nitrate hexahydrate (Zn­(NO_3_)_2_·6H_2_O), 2-methylimidazole (2-MeIm), ferrous chloride (FeCl_2_), ethylenediamine, citric acid, acrylamide (AAm), *N*,*N*′-methylenebis­(acrylamide) (MBAA), tetramethylethylenediamine (TEMED), ammonium persulfate (APS), lithium chloride (LiCl), methylene blue, and fluorescent carboxylate-modified polystyrene microbeads (yellow-green, 2 μm diameter) were obtained from commercial suppliers Sigma-Aldrich. Lithium was obtained from ACROS Organics, Fisher Scientific, and used as received without further purification.

### Synthesis of Fe_3_O_4_ Magnetic Nanoparticles

Fe_3_O_4_ MNPs were synthesized by adapting an established FeCl_2_-based route.^43^ An aqueous FeCl_2_ solution (0.05 mol/L) was prepared and adjusted to pH 11.3 with ethylenediamine. The mixture was stirred vigorously for 24 h (magnetic stirrer). Citric acid (1.5 g/mL) was then added, and the mixture was stirred for 2 h to improve colloidal stability in deionized (DI) water. MNPs were collected with permanent magnets, the supernatant was decanted, and the particles were washed with DI water three times using the same magnetic separation. The mean particle diameter was 55 nm as measured by TEM (Figure S1). The carboxyl group on the MNP surfaces remains stable over several months.^44^

### Synthesis of MZIF-L and MZIF-L Chain Clusters

The ZIF-L synthesis procedure was modified with adjusted parameters in preliminary experimental procedures.^40^ An aqueous dispersion containing 0.1 wt % MNPs and zinc nitrate hexahydrate (0.5 mol/g) was vigorously mixed for 30 min. A 2-methylimidazole aqueous solution (2 mol/g) was added to the mixture and mixed by hand-shaking for 1 min. The total solution volume was 20 mL, and the precursor ratio of Zn:2MeIm was 2:8.

To synthesize isotropic MZIF-L, the total solution was mounted on a tube rotator (Rotamix, ATR) at 15 rpm to prevent MNP sedimentation during growth. For the anisotropic MZIF-L clusters, the mixture was poured into an 80 mm diameter Petri dish and positioned between two permanent magnets with a 4 in. diameter. Magnetic flux density (B) was controlled by adjusting the distance between the magnets. After 24 h, the resultant MZIF-L was magnetically collected and washed with DI water three times to remove unreacted chemicals and unattached ZIF microparticles by carefully decanting the supernatant each time. These purification steps were repeated three times.

### Synthesis of PAAm/MZIF-L Chain Cluster Composite Hydrogels

Composite hydrogels were prepared by dissolving AAm and LiCl in DI water with 0.2 wt % MZIF-L chain clusters. The target concentrations of AAm monomer and LiCl dispersed in DI water were 2.17 and 2 M, respectively.^45^ MBAA (cross-linker, 0.13 wt % relative to AAm), APS (initiator, 0.16 wt %, relative to AAm), and TEMED (accelerator, 0.3 wt %, relative to AAm) were added sequentially after complete dissolution. The precursor was cast into a glass cell with a 400 μm spacer gap and placed between two permanent magnets at an angle to induce targeted alignment of MZIF-L inside the hydrogel (0°, 45°, 90°). The magnet setup was the same setup with MZIF-L chain cluster synthesis, but the setup was rotated 90° to put the glass cells into the setup (Figure S13). After 2 h of steady magnetic setup, the PAAm/MZIF-L composite hydrogels were demolded from the cell.

### Characterization

Scanning electron microscopy (SEM) imaging was performed on a Hitachi S-3400N. Transmission electron microscopy (TEM) was performed on a Hitachi HT7700. Optical microscopy was conducted using an Olympus BX51. Confocal laser scanning microscopy (CLSM) used the same microscope with a 500 nm emission filter.

### Magnetic Rotation of MZIF-L Chain Clusters

Rotational actuation experiments were carried out on a custom-made magnetic stirrer equipped with a single permanent magnet (Figure S14). Rotation rates were 5, 50, 100, and 200 rpm at an applied B = 0.03 T. MZIF-L chain clusters were dispersed in DI water and introduced into a 400 μm gap glass cell. Methylene blue (TCI) was used for the mixing demonstrations.

## Supplementary Material









## Notes

TEM images of magnetic nanoparticles, images of the custom magnetic setup for MZIF-L chain clusters and their magnetic rotation, and hydrogel composites, optical micrographs of MNP chains and MZIF-L clusters, SEM micrographs of ZIF-L/MZIF-L/MZIF-L chain clusters synthesized with various magnetic flux densities, mechanical property, redispersal procedure of MZIF-L chain clusters after magnetic rotation, and figure for dye mixing using MZIF-L chain clusters (PDF)
Rotational motion of MZIF-L chain clusters dispersed in aqueous medium with different rotation rates, applied magnetic flux density: 30 mT, and MZIF-L chain clusters synthesized under 50 mT (MP4)
Redispersal of MZIF-L chain clusters by applying mild agitation, applied magnetic flux density: 30 mT, and MZIF-L chain clusters synthesized under 50 mT. (MP4)
Blue dye mixing with rotational MZIF-L chain clusters dispersed in aqueous medium, applied magnetic flux density was 30 mT, rotation rate was 200 rpm, and MZIF-L chain clusters synthesized under 50 mT (MP4)
