# Supplementary material for: Assembling Magneto-Responsive Metal–Organic Framework Long-Range Chains
Source: ACS Appl Mater Interfaces. 2025 Dec 3;17(50):68473–81. doi: 10.1021/acsami.5c19158 (PMC12723633; doi:10.1021/acsami.5c19158)
Supplement: Supplementary file 1 [file am5c19158_si_001.pdf]

Supporting information

Assembling Magneto-Responsive Metal-Organic Framework Long-range Chains

Jisoo Jeon[1], Valeriia Poliukhova[1], Hannah Y Cook[1], Vladimir V. Tsukruk[1]*

[1]School of Materials Science and Engineering, Georgia Institute of Technology, Atlanta, GA 30332, USA

*Corresponding author e-mail: vladimir@mse.gatech.edu

Video S1. Rotational motion of MZIF-L chain clusters dispersed in aqueous medium with different rotation rates. Applied magnetic flux density: 30 mT. MZIF-L chain clusters synthesized under 50 mT.

Video S2. Redispersal of MZIF-L chain clusters by applying mild agitation. Applied magnetic flux density: 30 mT. MZIF-L chain clusters synthesized under 50 mT.

Video S3. Blue dye mixing with rotational MZIF-L chain clusters dispersed in aqueous medium. Applied magnetic flux density was 30 mT. Rotation rate was 200 rpm. MZIF-L chain clusters synthesized under 50 mT.

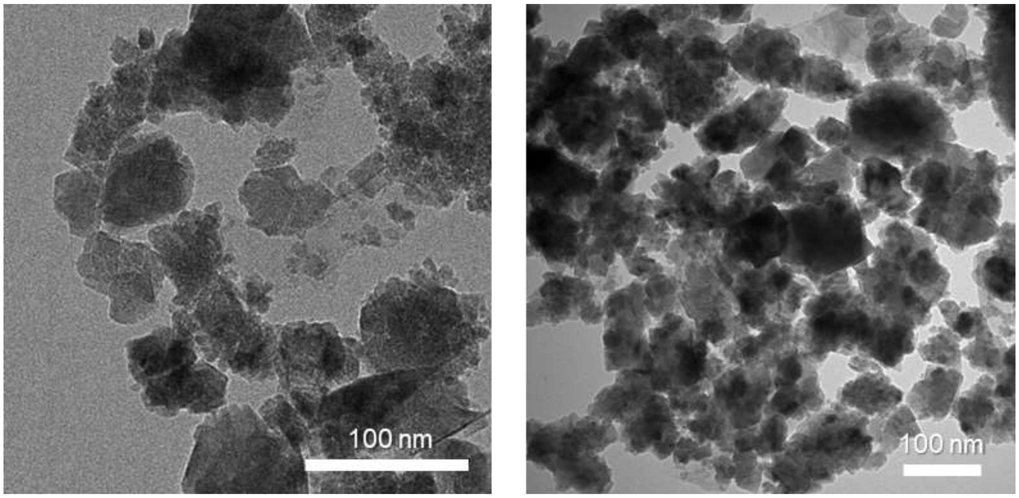

Figure S1. TEM micrographs of magnetic nanoparticles.

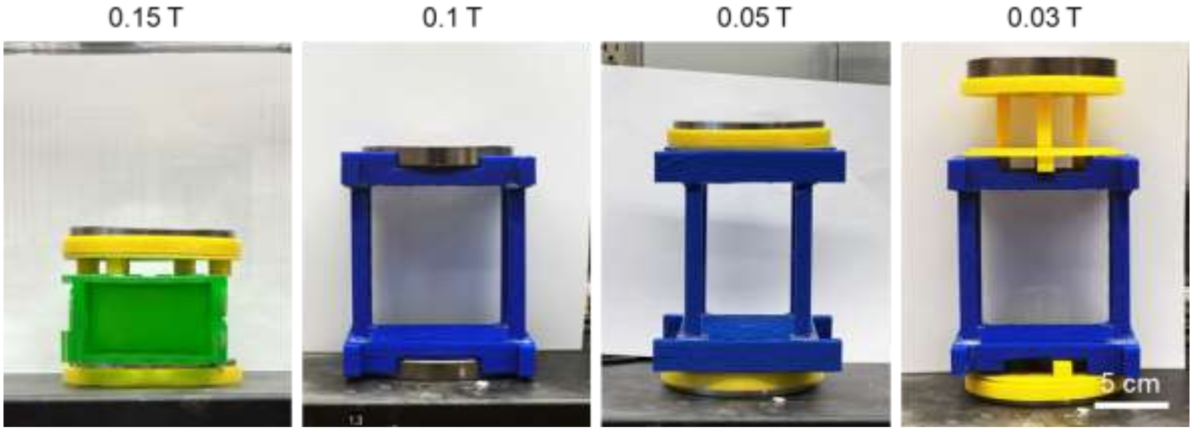

Figure S2. Two permanent magnets on a 3D-printed stage with a controllable gap as controlled by yellow parts).

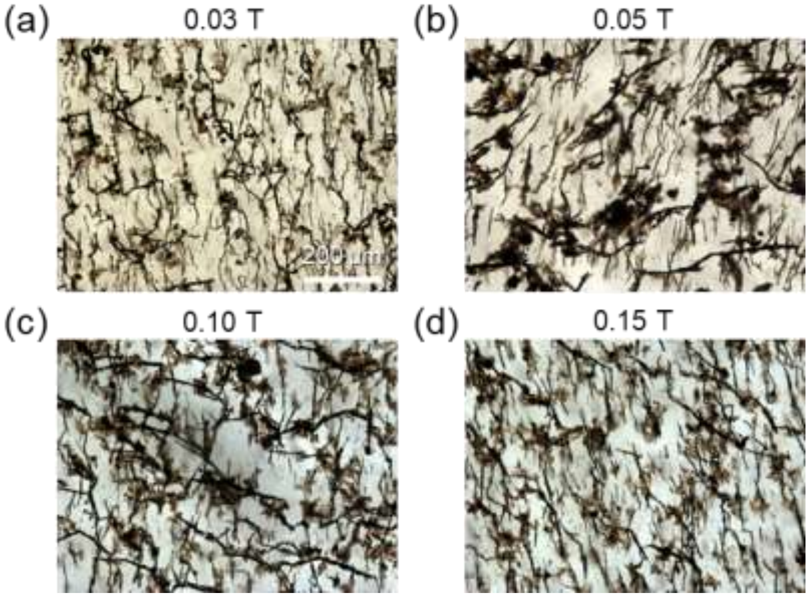

Figure S3. Optical micrographs of chain-like MNPs under different magnetic fields. Applied magnetic flux densities are: (**a**) 0.03 T, (**b**) 0.05 T, (**c**) 0.10 T, and (**d**) 0.15T. Scale bar on (a) shares with (b-d).

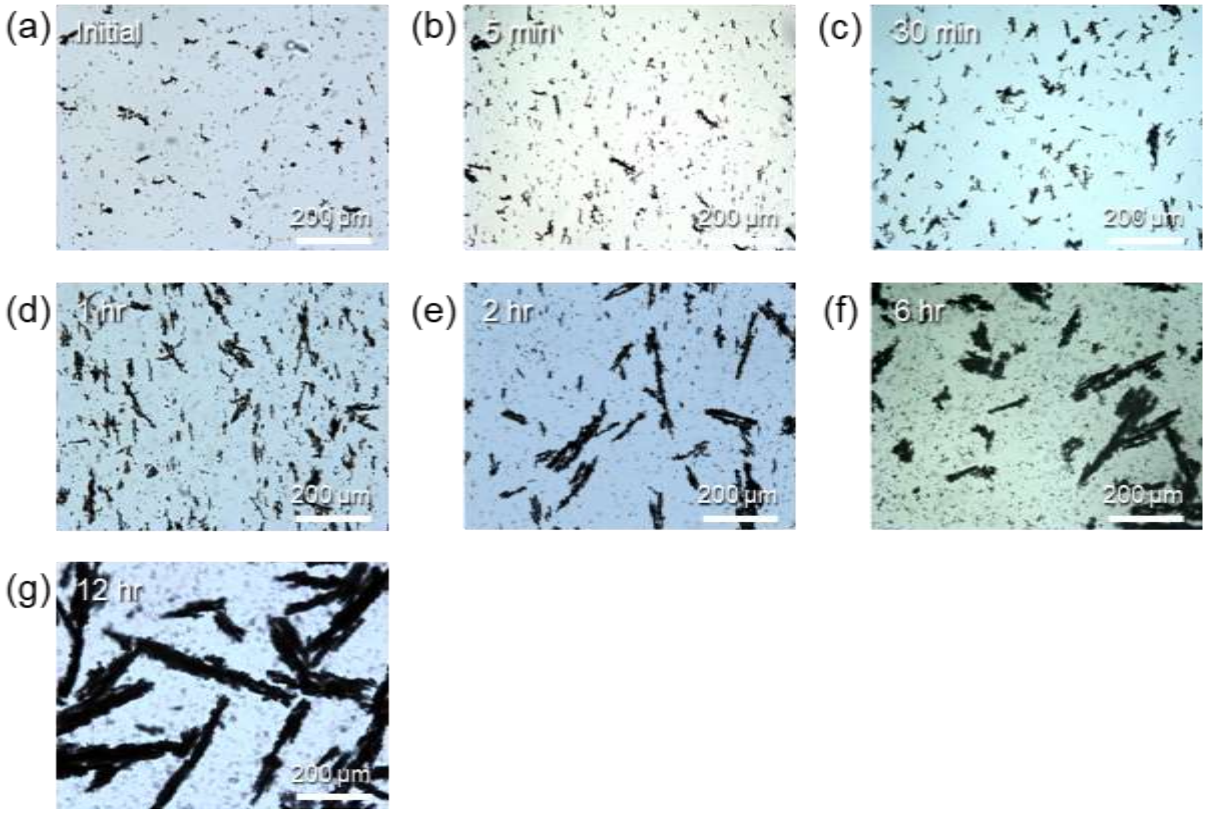

Figure S4. Optical micrographs of anisotropic MZIF-L with different processing times. Applied magnetic flux density is 0.05 T.

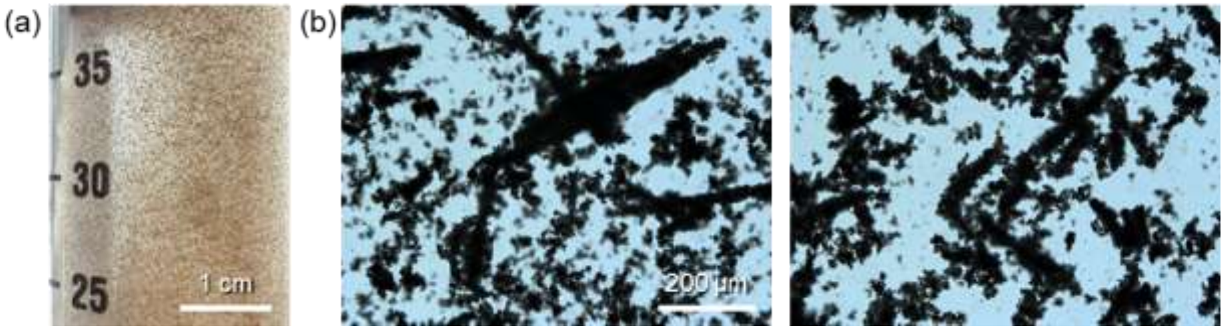

Figure S5. (a) Photo and (b) optical micrographs of MZIF-L chain clusters after 5 months of synthesis.

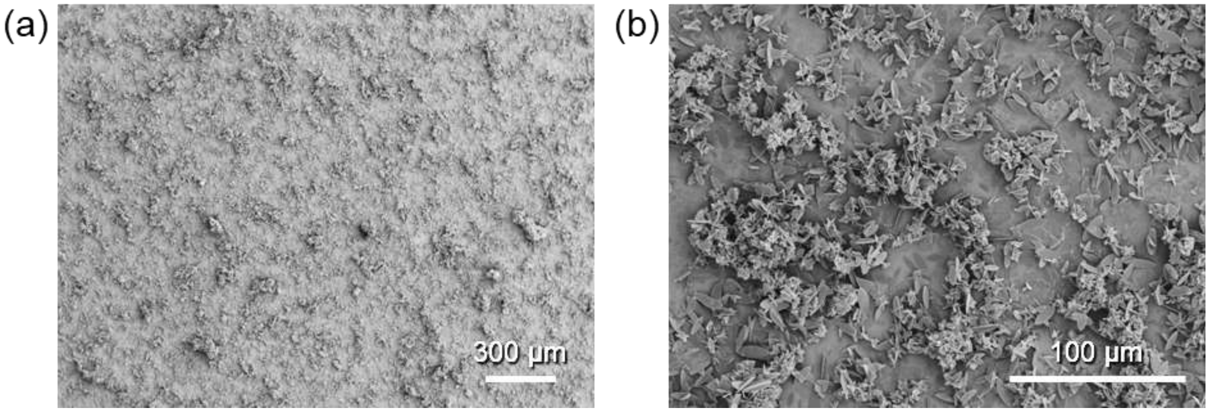

Figure S6. SEM micrographs of MZIF-L chains and their aggregates at lower magnifications.

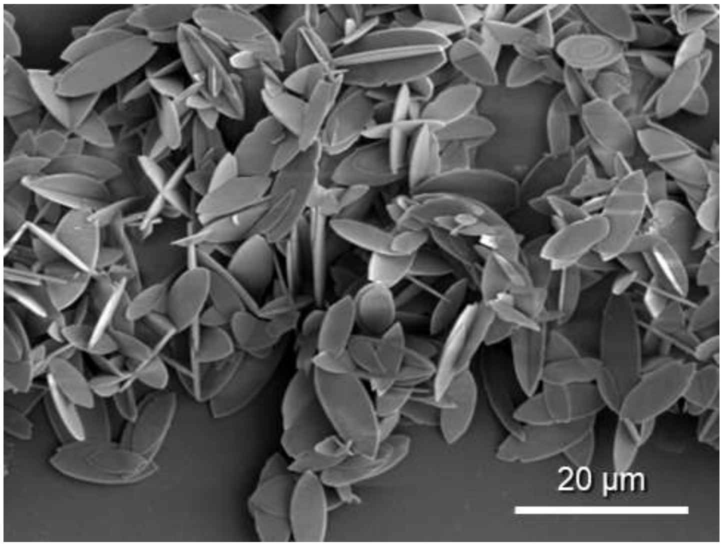

Figure S7. SEM micrograph of ZIF-L, synthesized without magnetic field and MNPs at higher magnification.

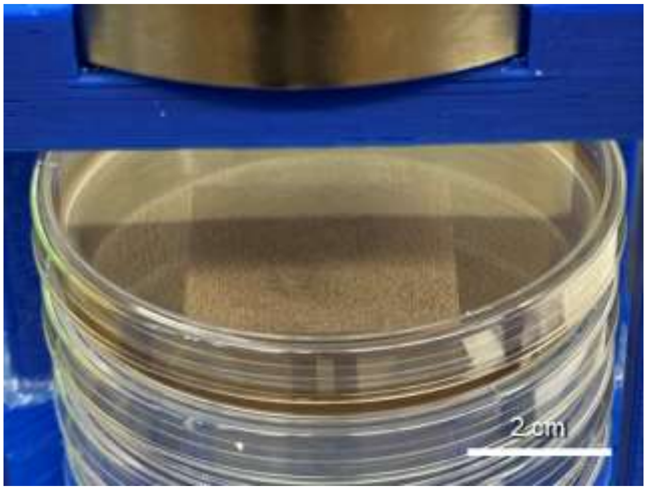

Figure S8. Photo of MNP chains in presence of magnetic field. Applied magnetic flux density was 0.1 T.

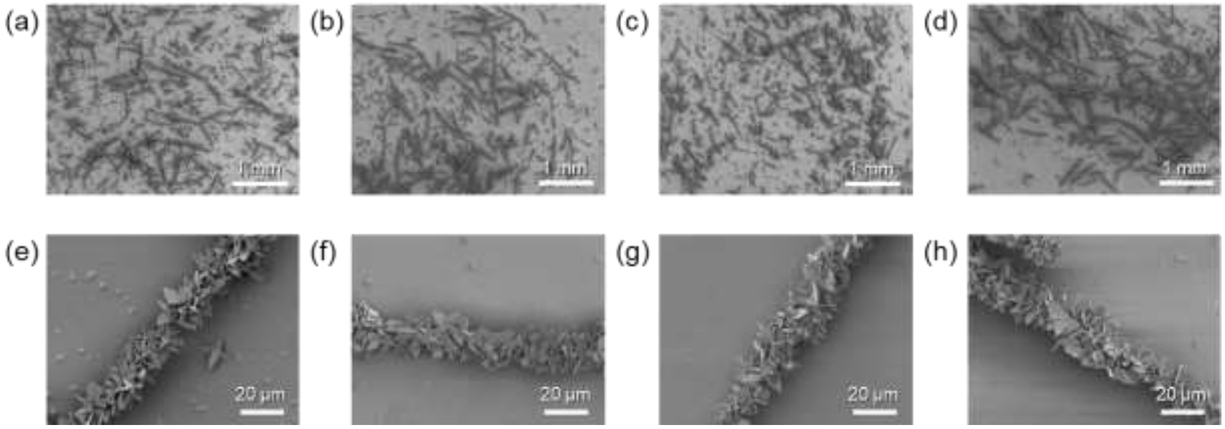

Figure S9. SEM micrographs of anisotropic MZIF-L clusters. Applied magnetic flux: (**a, e**) 0.03 T, (**b, f**) 0.05 T, (**c, g**) 0.1 T, and (**d**, **h**) 0.15T.

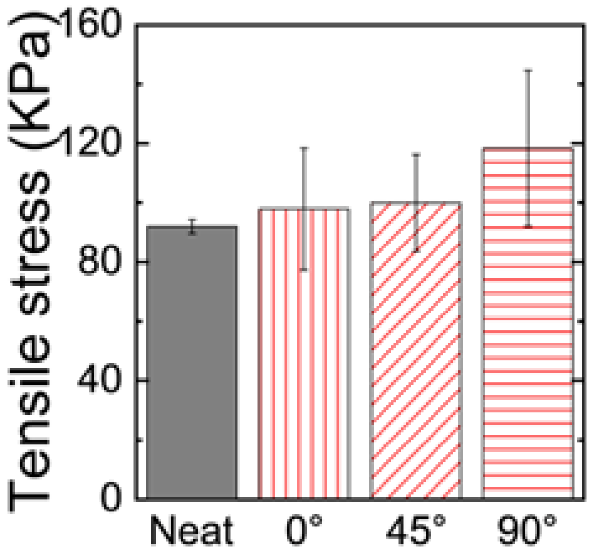

Figure S10. Summarized tensile stress of AAm/MZIF-L chain clusters composite hydrogel.

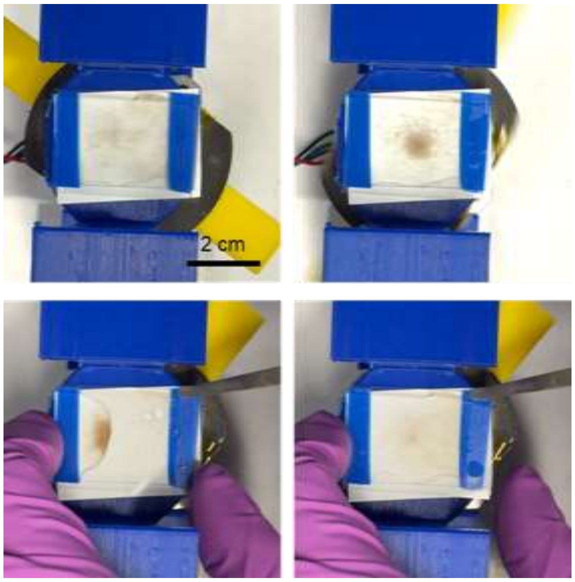

Figure S11. Redispersal of MZIF-L chain clusters by applying mild agitation.

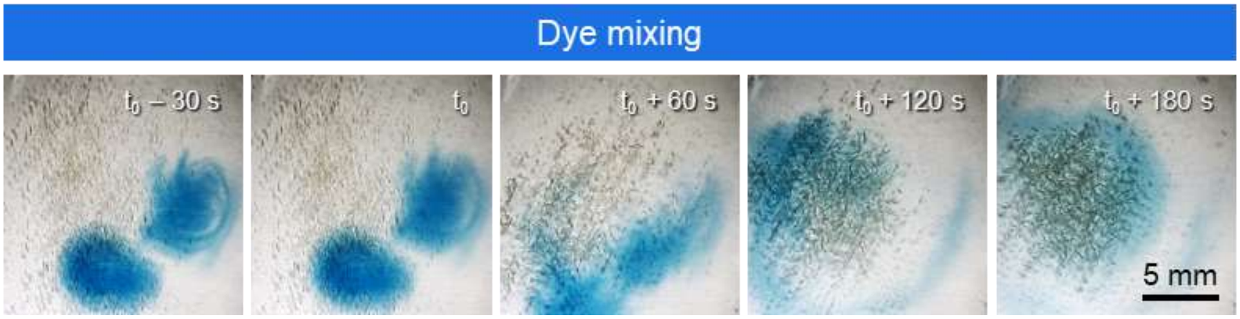

Figure S12. Dye mixing through the rotation of anisotropic MZIF-L clusters. MZIF-L clusters were dispersed aqueous medium and injected to 400 μm gap glass cell. Applied magnetic flux density was 30 mT. Rotation rate is 300 rpm.

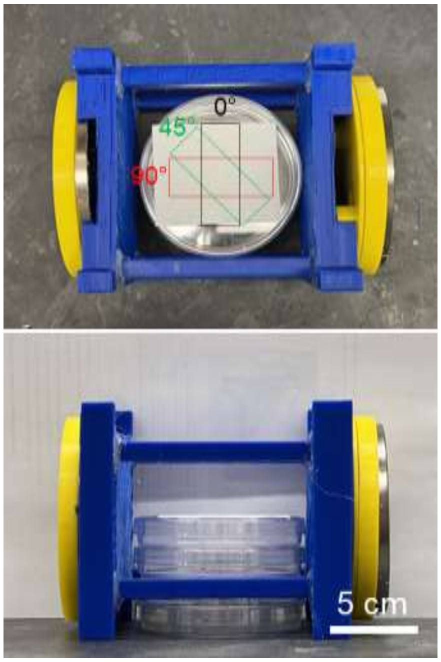

Figure S13. Magnetic setup to arrange MZIF-L chain clusters in PAAm/MZIF-L chain cluster composite. The boxes in the photo indicate location of glass cell.

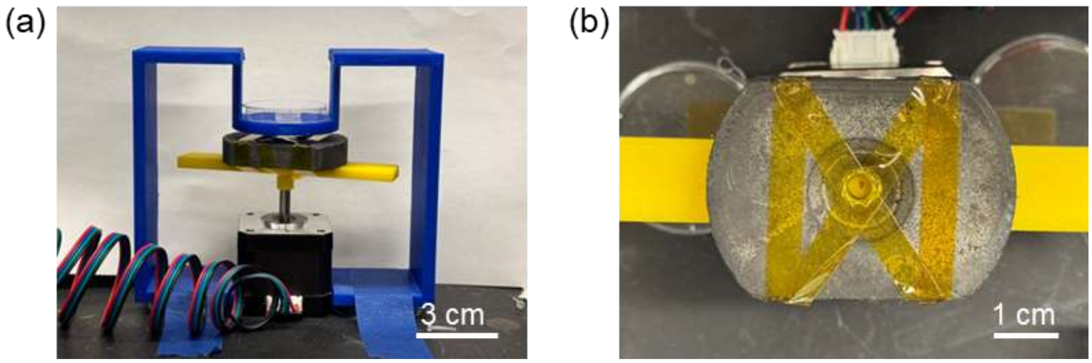

Figure S14. (a) Custom magnetic setup to regulate direction of rotation. (b) Shape of permanent magnet in top view.
